# The relationship between systemic therapies and low skeletal muscle mass in patients with intermediate and advanced hepatocellular carcinoma

**DOI:** 10.3389/fimmu.2025.1557839

**Published:** 2025-03-05

**Authors:** Jingjing Chen, Xueying Huang, Qiaoxin Wei, Songtao Liu, Wenyan Song, Mei Liu

**Affiliations:** ^1^ Department of Oncology, Beijing You’an Hospital, Capital Medical University, Beijing, China; ^2^ Department of Imaging, Beijing You’an Hospital, Capital Medical University, Beijing, China

**Keywords:** hepatocellular carcinoma, systemic therapy, immunotherapy, targeted immunotherapy combination treatment, targeted therapy, low skeletal muscle mass

## Abstract

**Background:**

Low skeletal muscle mass (LSMM) has been associated with poor prognosis in hepatocellular carcinoma (HCC) patients receiving systemic therapy. However, its impact across different treatment regimens remains unclear.

**Methods:**

A retrospective study analyzed 714 patients with intermediate and advanced HCC, divided into immunotherapy (I, n=85), target-immunotherapy combination (I+T, n=545), and targeted therapy (T, n=84) groups based on treatment. Skeletal muscle was assessed via computed tomography (CT) at the third lumbar vertebral level (L3) before and after 3 months of treatment. LSMM was evaluated by the third lumbar skeletal muscle index (L3-SMI) using a predefined threshold. Patients were stratified by baseline values and treatment changes. Kaplan-Meier and Cox models were used to compare overall survival (OS) and progression-free survival (PFS).

**Results:**

There was no significant difference in the loss of muscle mass among the three groups of LSMM patients; whereas, non-LSMM(NLSMM) patients in group T lost more muscle mass than those in group I (P=0.040).In the I+T group, patients who achieved an objective response (ORR) had less muscle mass loss than those without (P=0.013), while the changes in muscle mass for patients in the I group and T group were unrelated to treatment response. Baseline or post-treatment LSMM was associated with poorer median OS, especially in the I+T group. Progressive LSMM was linked to shorter median PFS (4.9 *vs* 5.7 months) and OS (9.8 *vs* 16.5 months), with similar results in the I+T group (mPFS, 4.2 *vs*. 5.8 months; mOS, 9.7 *vs* 16.1 months). Patients with LSMM had a higher incidence of treatment-related SAEs, particularly ascites and fatigue.

**Conclusion:**

In patients with combined LSMM in hepatocellular carcinoma, muscle loss did not significantly differ between those treated with I, I+T, and T; however, T treatment contributed to muscle mass loss in NLSMM patients. Greater muscle loss correlated with poorer treatment outcomes and increased SAEs, and baseline, post-treatment, and progressive LSMM were linked to significantly worse prognoses, particularly with combined treatment regimens.

## 1 Introduction

Liver cancer is the sixth most common malignant tumor worldwide and the third leading cause of cancer-related deaths globally. Hepatocellular carcinoma (HCC) is the most prevalent type of primary liver cancer, accounting for approximately 75%-85% of all liver cancer cases (1). Due to the difficulty in early diagnosis and rapid disease progression, the majority of patients are diagnosed at intermediate to advanced stages, where surgical intervention is no longer an option. For these patients, systemic therapy is the primary treatment choice that offers survival benefits and can be categorized into three types: targeted drug therapy, immunotherapy, and combined treatment strategies (2, 3). Immune checkpoint inhibitors (ICIs) include agents targeting programmed cell death 1 (PD-1) and programmed cell death ligand 1 (PD-L1); molecular-targeted therapies primarily consist of multikinase inhibitors (TKI) and more specific small molecule agents, such as anti-vascular endothelial growth factor (VEGF) inhibitors and fibroblast growth factor receptor 4 (FGFR4) inhibitors, etc. Combined treatment strategies mainly involve the co-administration of molecular targeted drugs with ICIs (4). Targeted immunotherapy combinations (Atezolizumab + bevacizumab or Durvalumab + tremelimumab) are preferred for first-line treatment, while monotherapy with targeted drugs can be considered for patients with contraindications to ICIs, and dual immunotherapy or monotherapy with immunotherapy can be chosen for patients at high risk of bleeding (5).

The long-term prognosis of HCC patients is related to various factors. In addition to liver function reserve, tumor staging, and treatment methods, maintaining nutritional balance and physical capacity are also important factors in improving the prognosis of advanced HCC patients (6). Sarcopenia is a disease characterized by the loss of muscle mass and a decline in physical function (7). Studies have shown that sarcopenia, characterized by low skeletal muscle mass(LSMM) as defined by computed tomography(CT),independently predicts the overall survival (OS) and progression-free survival (PFS) of HCC patients (8–10). In the systemic treatment of unresectable hepatocellular carcinoma(uHCC), LSMM is associated with adverse clinical outcomes of TKI such as sorafenib and lenvatinib. Similarly, in patients receiving immunotherapy, most studies report that patients with LSMM have poorer OS and PFS (10). Currently, many studies focus on the relationship between LSMM and the prognosis of hepatocellular carcinoma (6–10), however, there is a lack of research on the role of different systemic treatments in skeletal muscle mass loss in patients with mid-to-advanced HCC, as well as the impact of changes in muscle mass on patient prognosis.

Therefore, this study aims to assess the impact of immunotherapy (I), combined immunotherapy and targeted therapy (I+T), and targeted therapy (T) on LSMM in patients with mid-to-advanced HCC, as well as to investigate the relationship between changes in skeletal muscle mass during treatment and treatment response and prognosis.

## 2 Materials and methods

### 2.1 Patients

A retrospective cohort study was conducted on HCC patients who received systematic treatment at Beijing You’an Hospital, affiliated with Capital Medical University, from January 2018 to February 2024. Inclusion criteria: (1) Patients aged ≥18 years; (2) Patients with Child-Pugh Class A or B, and some Child-Pugh C patients from trials on systemic treatment safety and efficacy across liver function levels; (3) Patients with mid-to-advanced HCC who received systematic treatment; (4) Patients who underwent CT assessment within one month before treatment. Patients with any of the following conditions were excluded: (1) Patients without abdominal computed tomography (CT) at baseline and three months after treatment; (2) Patients with other malignancies or severe extrahepatic organ-based diseases; All patient data were retrieved from electronic medical records. A total of 714 cases were included in the final data analysis. Follow-up for all patients continued until August 31, 2024. The study protocol complied with the ethical guidelines of the Declaration of Helsinki, was approved by the Institutional Review Board of Beijing You’an Hospital(LL-2022-027-K), and exempted from the requirement for informed consent since all data were analyzed retrospectively and anonymously.

### 2.2 Treatment grouping and tumor response assessment

Patients were divided into three groups based on treatment regimens: Immunotherapy (I), Combined Therapy (I+T), and Targeted Drug Therapy Group (T). ICIs include inhibitors targeting Programmed Cell Death 1 (PD-1) (sintilimab, tislelizumab, camrelizumab, pembrolizumab) and Programmed Cell Death Ligand 1 (PD-L1) inhibitors (atezolizumab, durvalumab); the T group mainly consists of multi-kinase inhibitors (TKI) (lenvatinib, regorafenib, donafenib, apatinib, sorafenib) and anti-vascular endothelial growth factor (VEGF) inhibitors (bevacizumab and its biosimilars). The combined therapy mainly involves the treatment of molecular targeted drugs in combination with ICIs. All anticancer drugs used in this study, including targeted therapies and immune checkpoint inhibitors, are covered by the China National Reimbursement Drug List (NRDL) or commercial medical insurance drug formularies, ensuring partial or full reimbursement under China’s medical insurance system and commercial health insurance plans.

In our institution, intermediate and advanced-stage HCC treatment is guided by clinical guidelines, tumor staging, Child-Pugh classification, and patient comorbidities. The choice of anticancer drugs is guided by the Barcelona Clinic Liver Cancer (BCLC) staging system, National Health Commission Guidelines for the Diagnosis and Treatment of Primary Hepatocellular Carcinoma, and National Comprehensive Cancer Network Clinical Practice Guidelines in Oncology (NCCN Guidelines). First-line options include atezolizumab with bevacizumab, sintilimab with bevacizumab biosimilar, or lenvatinib monotherapy, among others. For patients with contraindications to combination therapy, single-agent treatment (targeted therapy or immunotherapy) is considered. Drug selection is based on availability, patient tolerance, and insurance coverage.

The treatment strategy for all intermediate and advanced HCC patients considers liver function, including cirrhosis staging. Management of underlying cirrhosis follows clinical guidelines, incorporating alcohol cessation, antiviral therapy (HBV/HCV), nutritional support (albumin supplementation), hepatoprotective drugs, and monitoring for complications such as ascites, esophageal varices, and hepatic encephalopathy. Liver function is closely monitored throughout treatment.

Tumor response was assessed using the modified Response Evaluation Criteria in Solid Tumors (mRECIST) after three months of treatment, with key indicators including the objective response rate (ORR), representing the proportion of patients with complete response (CR) and partial response (PR) (11). OS (Overall Survival) and PFS (Progression-Free Survival) were calculated from the start of systematic treatment.

### 2.3 Imaging analysis

All patients underwent abdominal CT scans within one month before treatment and three months after treatment. The CT scans were performed using a US LightSpeed VCT CT 64 scanner. Skeletal Muscle Area (cm²): The cross-sectional area (cm²) of skeletal muscles at the third lumbar vertebra (L3) on CT imaging was used to estimate body skeletal muscle mass, including the psoas major, erector spinae, quadratus lumborum, transversus abdominis, internal oblique, external oblique, and rectus abdominis muscles. The axial images at the L3 level were manually measured on a dedicated workstation (SliceOmatic software, version 5.0) for specific tissues (−29 to +150 Hounsfield units (HU) threshold). The total skeletal muscle area of the L3 cross-section was independently assessed by two radiologists. In case of disagreement, a third doctor intervened and a consensus was reached. The Skeletal Muscle Index (SMI) was calculated as the L3 level skeletal muscle area (cm²) divided by the square of height (m²) to obtain the L3-SMI. An L3-SMI value less than 42 cm²/m² for males and less than 38 cm²/m² for females is considered to have low skeletal muscle mass(LSMM) (12). We assessed only muscle mass, excluding muscle function assessments.

### 2.4 Clinical data

Baseline data of patients were collected, including age, gender, BMI, etiology, history of previous treatments, BCLC staging, ECOG PS, portal vein tumor thrombus (PVTT), extrahepatic metastasis, and Child-Pugh classification. Laboratory data were also collected during the follow-up period, including complete blood count, liver function (total bilirubin, albumin), coagulation, and alpha-fetoprotein (AFP) related indicators. The relative change (%) throughout the entire treatment process was calculated as follows: ΔSMI = (SMI at 3 months - baseline SMI)/baseline-SMI * 100%, with the threshold defined as 10%. Progressive LSMM is defined as a decrease in ΔSMI > 10% (13). Body Mass Index (BMI) was calculated using the formula: BMI = weight (kg)/height squared (m²).

### 2.5 Statistical analysis

Statistical analysis and graphical production were conducted using SPSS version 27.0 (IBM) and RStudio version 2024.09.0 + 375. A P-value of less than 0.05 was considered to indicate a statistically significant difference. Demographic data and disease characteristics of patients in the immunotherapy group, combined therapy group, and targeted therapy group were compared. Continuous variables are presented as mean ± standard deviation (SD), and categorical variables are presented as counts and percentages (%). The T-test was used for intergroup comparison of parametric data, while the Mann-Whitney-U test was used for non-parametric data. The chi-square test (χ2 test) and Kruskal-Wallis H test were used for intergroup comparison of categorical variables. Continuous variables with a normal distribution were compared across multiple groups using one-way ANOVA. Logistic regression analysis was used to study characteristics associated with LSMM. The Kaplan-Meier method was employed to estimate PFS and OS for each group and to plot survival curves. Subsequently, the Cox proportional hazards model was constructed to test for statistically significant differences in survival times between different groups. Patients lost to follow-up were censored at their last known follow-up date in survival analysis.

## 3 Results

### 3.1 Baseline characteristics

This study initially included 5,790 patients, of which 5,076 were excluded for various reasons. Ultimately, 714 patients were enrolled and divided into the immunotherapy group (I) with 85 patients, the combined therapy group (I+T) with 545 patients, and the targeted therapy group (T) with 84 patients. **Table 1** records the baseline characteristics of the entire cohort. The majority of patients were male (n=606, 84.9%), with an average age of 58.3 (SD ± 10.5) years and an average BMI of 23.92 ± 3.67 kg/m². Hepatitis B virus (HBV) infection was the main etiology in this cohort (n=676, 94.7%). Most patients had an ECOG-PS score of 1 (90.9%), Child-Pugh Class A (62.8%), were enrolled at the first-line treatment stage (64.9%), and were at BCLC Stage C at the start of treatment (71.9%), with distant metastasis present in 317 patients(44.4%). Previously, 98 (13.7%) and 566 (79.3%) patients had undergone surgical resection and transarterial chemoembolization (TACE) treatment, respectively. Among ICIs, sintilimab (37.8%), tislelizumab (24.8%), and camrelizumab (17%) were the most used, while the most common targeted therapies were lenvatinib (53.5%), bevacizumab (16%), and regorafenib (9.1%). Overall, the average SMI of the study patients was 43.43[38.65,49.9] cm²/m², with 273 (38.2%) diagnosed with LSMM. Radiological assessments showed CR in 53 cases (7.4%), PR in 155 cases (21.7%), SD in 260 cases (36.4%), and PD in 246 cases (34.5%). The objective tumor response rate was 29.1%.

**Table 1:** Baseline characteristics of hepatocellular carcinoma patients.

Variables	Total (n = 714)	I (n = 85)	I+T (n = 545)	T (n = 84)	*P*
Age, Mean ± SD	58.26 ± 10.45	61.47 ± 11.45	57.65 ± 10.13	58.98 ± 10.92	0.006
Gender(Male), n(%)	606 (84.87)	68 (80.00)	463 (84.95)	75 (89.29)	0.241
BMI, Mean ± SD	23.92 ± 3.67	23.60 ± 3.82	23.93 ± 3.57	24.19 ± 4.10	0.574
L3-SMI cm2/m2, M (Q_1_, Q_3_)	43.43 (38.65, 49.90)	42.84 (39.26,47.44)	43.43 (38.17,50.09)	44.46 (39.91,50.28)	0.163
LSMM,n(%)	273 (38.24)	33 (38.82)	212 (38.90)	28 (33.33)	0.616
HBV, n(%)	676 (94.68)	81 (95.29)	518 (95.05)	77 (91.67)	0.410
HCV, n(%)	111 (15.55)	12 (14.12)	84 (15.41)	15 (17.86)	0.786
Child-Pugh, n(%)					0.444
A	448 (62.75)	48 (56.47)	347 (63.67)	53 (63.10)	
B	234 (32.77)	31 (36.47)	177 (32.48)	26 (30.95)	
C	32 (4.48)	6 (7.06)	21 (3.85)	5 (5.95)	
BCLC, n(%)					0.274
B	169 (23.67)	22 (25.88)	123 (22.57)	24 (28.57)	
C	513 (71.85)	57 (67.06)	401 (73.58)	55 (65.48)	
D	32 (4.48)	6 (7.06)	21 (3.85)	5 (5.95)	
ECOG-PS, n(%)					0.890
0	18 (2.52)	2 (2.35)	13 (2.39)	3 (3.57)	
1	649 (90.90)	78 (91.76)	494 (90.64)	77 (91.67)	
2	47 (6.58)	5 (5.88)	38 (6.97)	4 (4.76)	
PVTT, n(%)	194 (27.17)	16 (18.82)	151 (27.71)	27 (32.14)	0.127
Metastasis, n(%)	317 (44.40)	30 (35.29)	250 (45.87)	37 (44.05)	0.188
Cirrhosis, n(%)	677 (94.82)	82 (96.47)	516 (94.68)	79 (94.05)	0.737
First-line, n(%)	463 (64.85)	45 (52.94)	360 (66.06)	58 (69.05)	0.043
Line, n(%)					0.054
1	464 (64.99)	46 (54.12)	360 (66.06)	58 (69.05)	
2	144 (20.17)	22 (25.88)	102 (18.72)	20 (23.81)	
3	106 (14.85)	17 (20.00)	83 (15.23)	6 (7.14)	
Pre-Surgery, n(%)	98 (13.73)	18 (21.18)	73 (13.39)	7 (8.33)	0.047
Pre-TACE, n(%)	566 (79.27)	66 (77.65)	427 (78.35)	73 (86.90)	0.183
Pre-Systemic therapy, n(%)	250 (35.01)	39 (45.88)	185 (33.94)	26 (30.95)	0.071
AFP≥ 400(ng/mL), n(%)	292 (40.90)	33 (38.82)	235 (43.12)	24 (28.57)	0.038
Tumor response in 3 months, n(%)					0.256
CR	53 (7.42)	6 (7.06)	42 (7.71)	5 (5.95)	
PD	246 (34.45)	34 (40.00)	185 (33.94)	27 (32.14)	
PR	155 (21.71)	10 (11.76)	121 (22.20)	24 (28.57)	
SD	260 (36.41)	35 (41.18)	197 (36.15)	28 (33.33)	
ORR, n(%)	208 (29.13)	16 (18.82)	163 (29.91)	29 (34.52)	0.057
DCR, n(%)	467 (65.41)	51 (60.00)	359 (65.87)	57 (67.86)	0.503

I, Immunotherapy; I+T, Targeted Therapy Combined with Immunotherapy; T, Targeted Therapy; P, P-value; BMI, Body Mass Index; L3-SMI, Third Lumbar Skeletal Muscle Index; LSMM, Low Skeletal Muscle Mass; HBV, Hepatitis B Virus; HCV, Hepatitis C Virus; BCLC, Barcelona Clinic Liver Cancer; ECOG-PS, Eastern Cooperative Oncology Group Performance Status; PVTT, Portal Vein Tumor Thrombosis; TACE, Transarterial Chemoembolization; AFP, Alpha-Fetoprotein; CR, Complete Response; PD, Progressive Disease; PR, Partial Response; SD, Stable Disease; ORR, Objective Response Rate; DCR, Disease Control Rate. Multiple group comparisons: ANOVA for normal data, Kruskal-Wallis test for non-normal data.

Among the 53 CR patients, as shown in **Supplementary Table 1**,most received first-line treatment (64.15%) and had a high prevalence of prior TACE treatment (90.57%). Additionally, 37.74% of patients had a history of surgery. Most patients had relatively preserved liver function, with Child-Pugh A (52.83%) and B (41.51%) classifications. The baseline BMI (24.24 ± 3.80) and L3-SMI (44.41 ± 6.91) were higher compared to all patients, with a low proportion of LSMM at 33.96%. The incidence of PVTT and distant metastasis was relatively low, at 7.55% and 26.42%, respectively. These characteristics suggest that CR patients generally have better baseline health status and lower tumor burden, which may contribute to achieving a complete response.

The median survival time for the entire cohort was 15.1 (95% CI, 13.6-16.7) months, and the median PFS was 5.6 (95% CI, 5.2 – 5.8) months. There were no significant differences in median survival and PFS among the three treatment groups. In Group I, 40 (47.1%) patients died, and 67 experienced progression (78.8%). The median survival time was 17.4 (95% CI: 12.1-22.7) months, and the median PFS was 4.5 (95% CI: 3.7 – 5.3) months. In Group I+T, 308 (56.5%) patients died, and 423 experienced progression (77.6%), with a median survival time of 14.9 (95% CI: 13.2-16.7) months and a median PFS of 5.6 (95% CI: 5.2 – 5.9) months. In Group T, 52 (61.9%) patients died, and 65 experienced progression (77.4%), with a median survival time of 15.6 (95% CI: 10.6-20.6) months and a median PFS of 6.0 (95% CI: 5.2 – 6.9) months.

### 3.2 Characteristics of LSMM in HCC patients

The characteristics of LSMM at baseline among HCC patients were first analyzed. Univariate regression showed that, compared with non-LSMM(NLSMM) patients, among the 273 HCC patients with LSMM, the majority were elderly (aged ≥60 years) (P<0.001), male (P<0.001), and received non-first-line treatment (P<0.001). Incorporating the aforementioned variables into a multivariate logistic regression model revealed that being elderly (aged ≥60 years) (OR=2.227, 95%CI 1.614–3.072), male (OR=2.653, 95%CI 1.713-4.111), and receiving non-first-line treatment (OR=0.465, 95%CI 0.335-0.646) were significantly associated with an increased likelihood of LSMM (P<0.001) (**Table 2**).

**Table 2:** Logistic regression analysis of factors associated with LSMM-baseline and LSMM in 3 months.

Variables	Baseline				3 months			
	Univariate		Mutlvariate		Univariate		Mutlvariate	
	OR (95%CI)	P	OR (95%CI)	P	OR (95%CI)	P	OR (95%CI)	P
Age≥60	2.405 (1.766-3.274)	<0.001	2.227 (1.614-3.072)	<0.001	2.162 (1.601-2.918)	<0.001	1.385 (0.890-2.156)	0.149
Gender (male)	3.029 (1.987-4.618)	<0.001	2.653 (1.713-4.111)	<0.001	3.118 (2.002-4.857)	<0.001	1.624 (0.863-3.056)	0.133
LSMM-baseline	/	/	/	/	28.054 (18.123-43.428)	<0.001	44.165 (26.559-73.442)	<0.001
Progressing LSMM	/	/	/	/	4.100 (2.788-6.029)	<0.001	12.013 (7.148-20.189)	<0.001
First-line	0.499 (0.364-0.683)	<0.001	0.465 (0.335-0.646)	<0.001	0.607 (0.446-0.828)	0.002	0.686 (0.431-1.091)	0.111

LSMM, Low Skeletal Muscle Mass; OR, Odds Ratio; CI, Confidence Interval; P, P-value.

After systematic treatment for 3 months, 338 patients were diagnosed with LSMM post-treatment, and they shared the same characteristics as patients with LSMM at baseline, predominantly being elderly (aged ≥60 years) (P<0.001), male (P<0.001), and receiving non-first-line treatment (P=0.002). Additionally, baseline LSMM (P<0.001) and the presence of progressive LSMM (P<0.001) were significantly associated with LSMM post-treatment. A multivariate logistic regression model identified baseline LSMM (OR=44.165, 95%CI 26.559–73.442) and the presence of progressive LSMM (OR=12.013, 95%CI 7.148–20.189) as independent risk factors for LSMM post-treatment (P<0.001) (**Table 2**).

Subsequently, subgroup analyses were conducted on the aforementioned variables. After 3 months of treatment, 44 (51.8%) patients in Group I, 262 (48.1%) in Group I+T, and 32 (38.1%) in Group T were diagnosed with LSMM. Univariate analysis showed that in all three groups, patients with LSMM post-treatment were associated with baseline LSMM (P<0.001) and the presence of progressive LSMM (I P=0.001, I+T P<0.001, T P=0.012). Additionally, LSMM patients in Group I were associated with non-first-line treatment (P=0.023), while those in Group I+T were predominantly aged ≥60 years (P<0.001), male (P<0.001), and receiving non-first-line treatment (P=0.011). Multivariate regression revealed that in all three groups, patients with LSMM post-treatment were significantly associated with baseline LSMM and the presence of progressive LSMM (P<0.001), consistent with the characteristics of the overall group of patients with LSMM (**Table 3**).

**Table 3:** Multifactorial logistic regression analysis of factors associated with LSMM 3 months in different groups.

Variables	I		I+T		T	
	OR (95%CI)	P	OR (95%CI)	P	OR (95%CI)	P
Age≥60	/	/	1.305 (0.769-2.215)	0.324	/	/
Gender(male)	/	/	1.823 (0.846-3.927)	0.125	/	/
LSMM-baseline	19.171 (5.138-71.538)	<0.001	55.683 (30.264-102.451)	<0.001	37.413 (8.645-161.910)	<0.001
Progressing LSMM	13.467 (2.780-65.238)	0.001	13.087 (7.136-23.998)	<0.001	12.352 (2.754-55.405)	0.001
First-line	0.373 (0.112-1.246)	0.109	0.699 (0.404-1.210)	0.201	/	/

I, Immunotherapy; I+T, Targeted Therapy Combined with Immunotherapy; T, Targeted Therapy; OR, Odds Ratio; P, P-value; LSMM, Low Skeletal Muscle Mass.

### 3.3 The relationship between muscle mass changes in LSMM and treatment regimens

**Figure 1:**
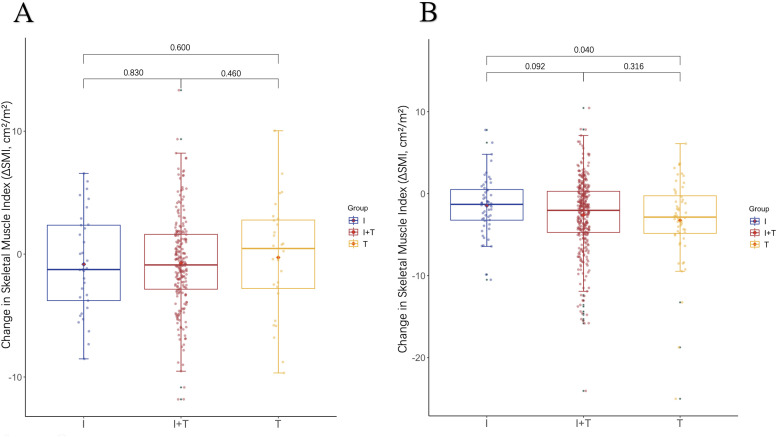
The difference in the change levels of ΔSMI after 3 months of treatment with three therapeutic regimens in patients with **(A)** LSMM and **(B)** NLSMM.LSMM,low skeletal muscle;NLSMM, non-low skeletal muscle.

**Figure 2:**
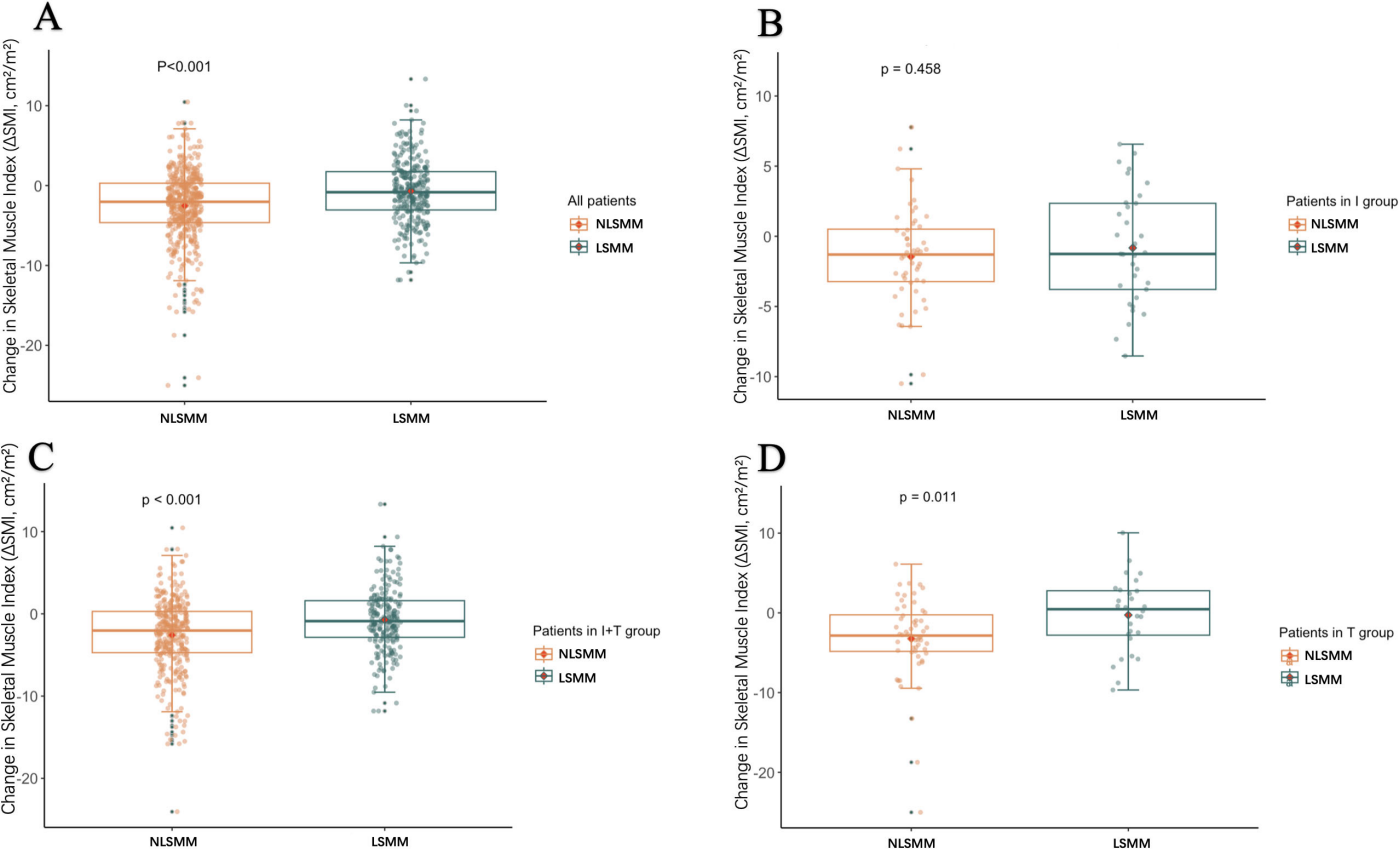
Box plots show the distribution of changes in ΔSMI after 3 months of treatment in patients with LSMM and those without LSMM: all patients **(A)**, immune group/I **(B)**, combined treatment group/I+T **(C)**, and targeted group/T **(D)**. ΔSMI, Skeletal Muscle Index; LSMM, low skeletal muscle.

We explored the relationship between LSMM, changes in skeletal muscle mass, and treatment regimens by analyzing the changes in SMI from baseline to 3 months after treatment, denoted as -ΔSMI: As shown in **Table 4**, there were no significant differences in baseline SMI and ΔSMI among the three groups of patients. Muscle loss did not significantly differ among patients with baseline LSMM across different treatment regimens. However, patients without baseline LSMM in Group I demonstrated less muscle loss compared to those in Group T (p=0.040), with the T regimen resulting in the greatest loss of muscle mass (**Figure 1**). As shown in **Table 5**, all LSMM patients exhibited less muscle loss than NLSMM patients (P<0.001), with the same results observable in Group I+T (P<0.001) and Group T (P=0.011) (**Figure 2**). This indicates that the I+T regimen and T regimen have an impact on muscle changes in patients with different baseline muscle conditions, while the I regimen does not have a significant effect.

**Table 4:** SMI and ΔSMI after 3 months of different groups.

Variables	I	I+T	T	Total	p
	n=85	n=545	n=84	n=714	
SMI (cm2/m2) Mean ± SD					
baseline	42.84 ± 7.87	44.25 ± 8.53	45.83 ± 8.34	44.27 ± 8.45	0.072
at 3 months	41.63 ± 8.11	42.39 ± 8.56	43.57 ± 8.10	42.44 ± 8.46	0.318
ΔSMI (cm2/m2) Mean ± SD					
in all patients	-1.21 ± 3.72	-1.86 ± 4.37	-2.25 ± 5.23	-1.83 ± 4.41	0.288
in LSMM patients	-0.83 ± 4.04	-0.73 ± 3.88	-0.28 ± 4.68	-0.69 ± 3.98	0.745
in NLSMM patients	-1.45 ± 3.52	-2.59 ± 4.52	-3.24 ± 5.25	-2.54 ± 4.53	0.111

SMI, Skeletal Muscle Index; ΔSMI, Change in Skeletal Muscle Index; I, Immunotherapy; I+T, Targeted Therapy Combined with Immunotherapy; T, Targeted Therapy; P P-value; SD, Standard Deviation; LSMM, Low Skeletal Muscle Mass.

**Table 5:** The impact of different treatment plans on ΔSMI in patients with different baseline muscle status (intra-group comparison).

ΔSMI (cm2/m2) Mean ± SD	LSMM		Total	p
	Yes	No		
total	-0.69 ± 3.98	-2.54 ± 4.53	-1.83 ± 4.41	p<0.001
I	-0.83 ± 4.04	-1.45 ± 3.52	-1.21 ± 3.72	p=0.458
I+T	-0.72 ± 3.88	-2.59 ± 4.52	-1.86 ± 4.37	p<0.001
T	-0.28 ± 4.68	-3.24 ± 5.25	-2.25 ± 5.23	p=0.011

SMI, Skeletal Muscle Index; ΔSMI, Change in Skeletal Muscle Index; I, Immunotherapy; I+T, Targeted Therapy Combined with Immunotherapy; T, Targeted Therapy; P, P-value; SD, Standard Deviation; LSMM, Low Skeletal Muscle Mass.

### 3.4 The relationship between changes in muscle mass and treatment response across different treatment regimens

**Figure 3:**
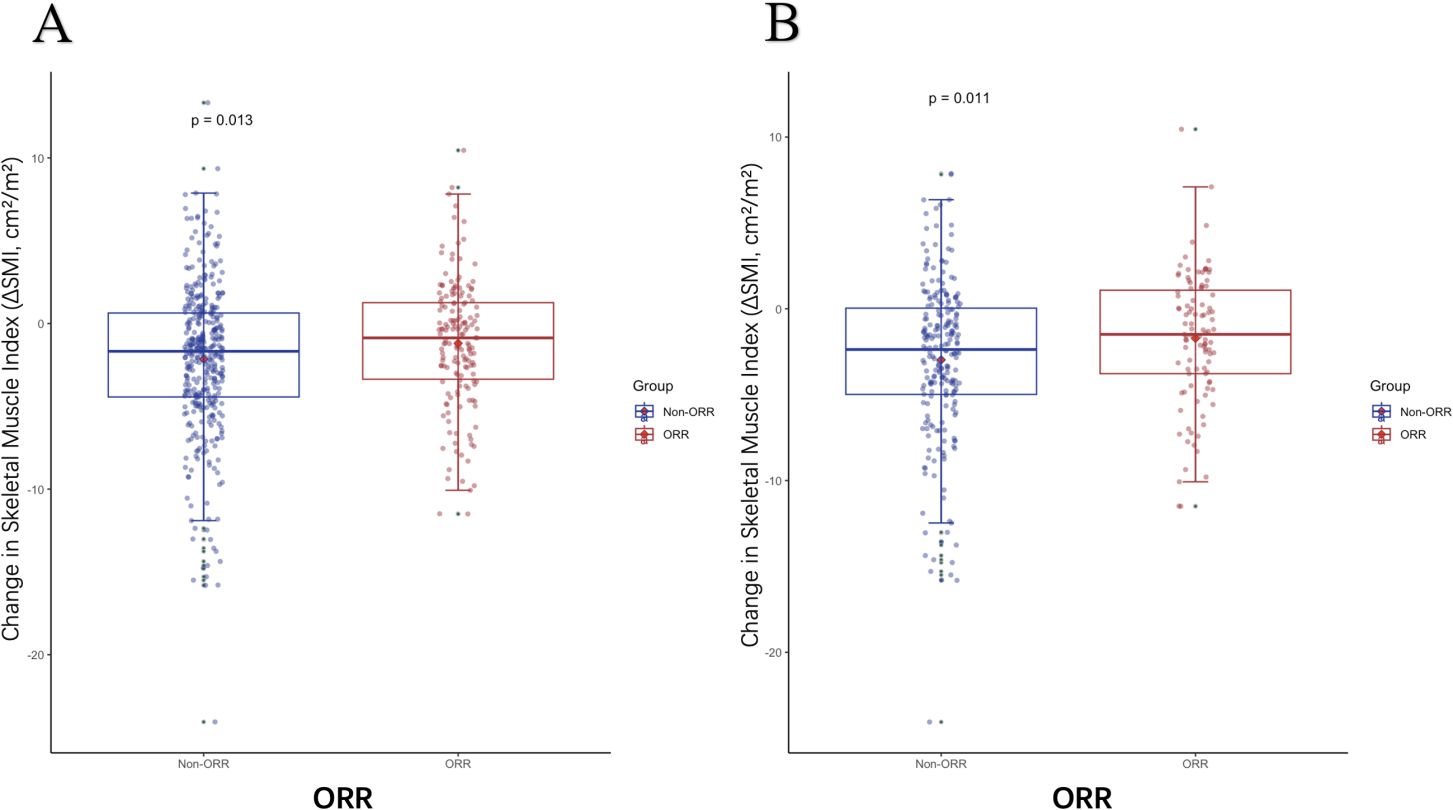
The difference in ΔSMI levels between patients with or without ORR. **(A)** The difference in ΔSMI between patients with or without ORR in the I+T group **(B)** The difference in ΔSMI between patients with or without ORR who are NLSMM in the I+T group; ORR, Objective Response Rate; NLSMM, non-low skeletal muscle; ΔSMI, the rate of change in Skeletal Muscle Mass Index; I, Immunotherapy; I+T, Targeted Therapy Combined with Immunotherapy; T, Targeted Therapy.

As shown in **Table 6**,there were no significant differences in baseline SMI between patients with or without objective response rate (ORR) in the entire cohort, but ORR was associated with the diagnosis of LSMM post-treatment (P=0.048). In the I and T groups, changes in muscle mass were not related to treatment response. However, in the I+T group, patients who achieved ORR had less muscle loss during treatment than those without treatment response (P=0.013), particularly among NLSMM patients(P=0.011)(**Figure 3**). In summary, patients receiving the combined treatment regimen who achieved ORR had significantly less muscle mass loss compared to those who did not respond to treatment.

**Table 6:** The relationship between changes in muscle mass and treatment response among different therapeutic regimens.

Variables	ORR-3months			
	0	1	total	P
SMI (cm2/m2) Mean ± SD	44.11 ± 8.49	44.66 ± 8.38	44.27 ± 8.45	0.431
LSMM-baseline	201 (39.6%)	72 (34.8%)	273 (38.2%)	0.226
LSMM-3months	252 (49.7%)	86 (41.5%)	338 (47.3%)	0.048
Progressing LSMM	125 (24.7%)	38 (18.4%)	163 (22.8%)	0.672
ΔSMI (cm2/m2) Mean ± SD	-2.03 ± 4.49	-1.34 ± 4.18	-1.83 ± 4.41	0.060
The relationship between ΔSMI and treatment response in patients with and without LSMM at baseline.				
LSMM patients	-0.85 ± 3.97	-0.24 ± 3.98	-0.69 ± 3.98	0.263
NLSMM patients	-2.80 ± 4.65	-1.93 ± 4.19	-2.54 ± 4.53	0.063
The relationship between ΔSMI and treatment response in patients under different therapeutic regimens.				
I	-1.17 ± 3.94	-1.37 ± 2.71	-1.21 ± 3.72	0.848
I+T	-2.15 ± 4.56	-1.19 ± 3.85	-1.86 ± 4.37	0.013
T	-2.30 ± 4.67	-2.16 ± 6.25	-2.25 ± 5.23	0.909
ΔSMI and treatment response in LSMM patients using different therapeutic regimens.				
I	-0.92 ± 4.10	-0.18 ± 4.21	-0.83 ± 4.04	0.736
I+T	-0.90 ± 3.93	-0.27 ± 3.75	-0.73 ± 3.88	0.293
T	-0.38 ± 4.32	-0.06 ± 5.64	-0.28 ± 4.68	0.872
ΔSMI and treatment response in NLSMM patients using different therapeutic regimens.				
I	-1.35 ± 3.86	-1.77 ± 2.12	-1.45 ± 3.52	0.724
I+T	-2.97 ± 4.76	-1.72 ± 3.81	-2.59 ± 4.52	0.011
T	-3.32 ± 4.59	-3.11 ± 6.41	-3.24 ± 5.25	0.888

SMI, Skeletal Muscle Index; ΔSMI, Change in Skeletal Muscle Index; I, Immunotherapy; I+T, Targeted Therapy Combined with Immunotherapy; T, Targeted Therapy; P, P-value; SD, Standard Deviation; LSMM, Low Skeletal Muscle Mass; ORR, Objective Response Rate; NLSMM, Non-Low Skeletal Muscle Mass.

### 3.5 Multivariate COX regression analysis of factors related to PFS and OS

**Figure 4:**
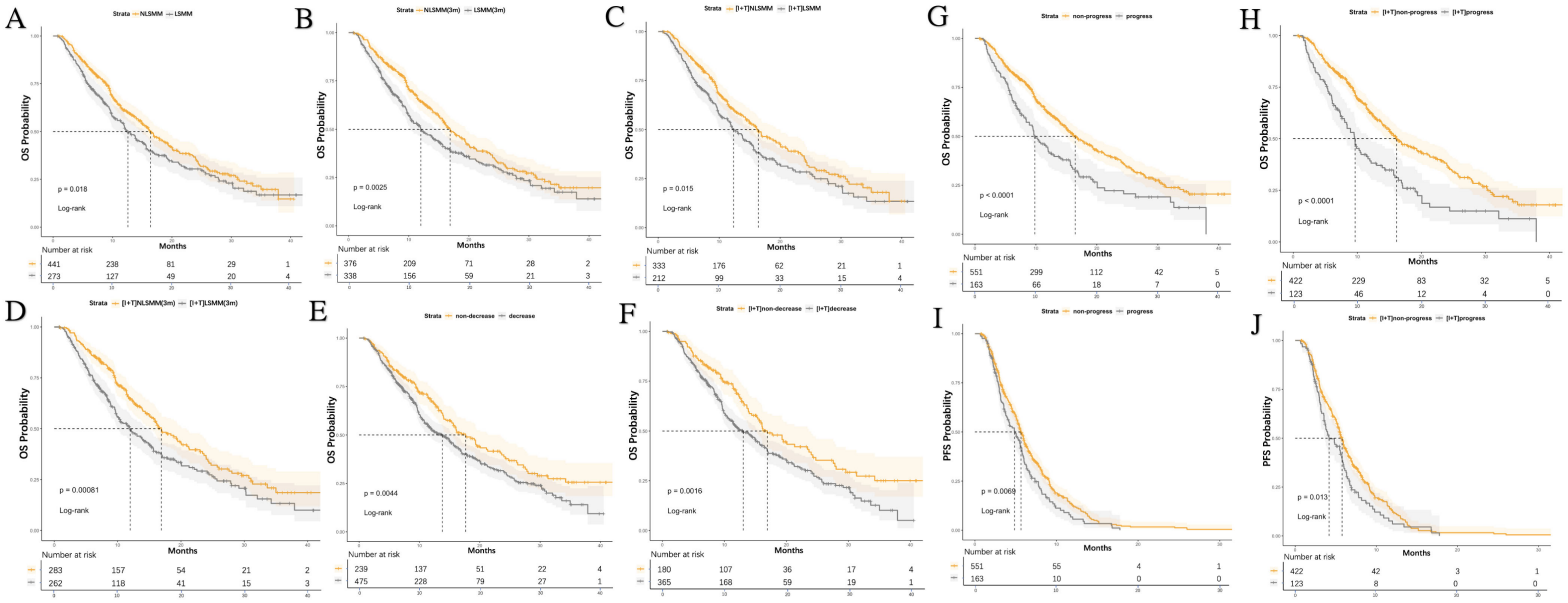
Kaplan-Meier curves for PFS and OS stratified by LSMM status, treatment group, SMI reduction, and progressive LSMM. **(A)** OS differences based on LSMM at baseline for all patients **(B)** OS differences based on LSMM status at 3 months; **(C)** LSMM at baseline in the I+T group **(D)** OS differences based on LSMM status at 3 months in the I+T group; **(E)** OS differences based on SMI reduction in all patients **(F)** OS differences based on SMI reduction in the I+T group; **(G)** OS differences based on progressive LSMM in all patients **(H)** OS differences based on progressive LSMM in the I+T group; **(I)** PFS differences based on progressive LSMM in all patients **(J)** PFS differences based on progressive LSMM in the I+T group; P-values were calculated using the log-rank test. PFS, progression-free survival; OS, overall survival; SMI, skeletal muscle index; LSMM, low skeletal muscle; NLSMM, non-low skeletal muscle; ΔSMI, the rate of change in Skeletal Muscle Mass Index; I, Immunotherapy; I+T, Targeted Therapy Combined with Immunotherapy; T, Targeted Therapy; 3m, 3months.

We stratified the clinical variables that might affect prognosis and used Kaplan-Meier analysis to explore their impact on PFS and OS. As of August 2024, 400 patients (56%) had died, and 555 patients (77.7%) had experienced progression. As shown in **Table 7**, among the overall patient population, the diagnosis of LSMM, both at baseline and post-treatment, was not associated with PFS. However, the median OS for patients with LSMM at baseline and post-treatment was significantly worse than for those without LSMM (baseline LSMM: median, 12.6 *vs*. 16.4, P = 0.018; post-treatment LSMM: median, 12.0 *vs*. 16.9, P = 0.002), with the same results observed in the I+T group (baseline LSMM: median, 12.4 *vs*. 16.4, P = 0.015; post-treatment LSMM: median, 12.0 *vs*. 16.9, P < 0.001) (**Figure 4**).

**Table 7:** Kaplan-Meier analysis of factors associated with PFS and OS in all patients.

Variables		PFS		OS	
		Median time 95% CI (months)	P	Median time 95% CI (months)	P
Baseline					
Age≥60		5.4 (5.1, 5.8)	0.008	14.9 (12.3, 17.1)	0.828
Gender (Male)		5.7 (5.4, 6.0)	0.410	15.6 (13.8, 17.4)	0.543
First line		5.5 (4.8, 6.1)	0.292	15.7 (13.9, 17.4)	0.774
PreSurgery		5.9 (4.7. 7.1)	0.211	15.2 (12.0, 18.4)	0.759
PreTACE		5.6 (5.3, 6.0)	0.208	16.1 (14.0, 18.1)	0.010
Metastasis		5.3 (4.7,5.9)	0.018	12.6 (10.5, 14.7)	0.009
PVTT		5.1 (4.4, 5.8)	0.083	10.4 (9.0, 11.9)	<0.001
AFP≥400ng/ml		5.6 (5.2, 6.0)	0.594	13.1 (11.0, 15.3)	0.127
Obesity (BMI≥24kg/m2)		5.6 (5.2, 5.9)	0.209	15.9 (13.8, 18.0)	0.990
Underweight (BMI ≤ 18.5kg/m2)		6.0 (5.4, 6.7)	0.514	15.0 (2.9, 27.2)	0.755
ECOG-PS	0	8.2 (6.1, 10.4)	0.377	20.1 (11.9, 28.4)	0.479
	1	5.6 (5.2, 5.9)		15.1 (13.4, 16.9)	
	2	4.8 (3.7, 5.9)		13.5 (8.9, 18.2)	
Child Pugh	A	5.6 (5.2, 6.0)	0.364	15.0 (13.2, 16.8)	0.759
	B	5.4 (4.9, 5.9)		15.6 (11.9, 19.3)	
	C	4.2 (2.4, 6.1)		15.9 (13.4, 18.3)	
BCLC	B	5.9 (5.5, 6.4)	0.309	19.3 (15.6, 22.9)	0.044
	C	5.4 (5.1, 5.8)		14.0 (12.1, 15.9)	
	D	4.2 (2.4, 6.1)		15.9 (13.4, 18.3)	
Treatment	I	4.5 (3.7, 5.3)	0.304	17.4 (12.1, 22.7)	0.417
	I+T	5.6 (5.2, 5.9)		14.9 (13.2, 16.7)	
	T	6.1 (5.2, 6.9)		15.6 (10.6, 20.6)	
Different therapies for patients with a baseline diagnosis of LSMM.					
LSMM-baseline		5.4 (5.0, 5.9)	0.217	12.6 (10.4, 14.7)	0.018
I		3.7 (2.8, 4.7)	0.279	17.4 (12.2, 22.7)	0.989
I+T		5.4 (4.8, 6.0)		16.4 (13.8, 19.0)	
T		6.2 (4.5, 7.9)		16.8 (11.0, 22.5)	
LSMM in I group		3.7 (2.8, 4,7)	0.270	17.4 (12.2, 22.7)	0.768
LSMM in I+T group		5.4 (4.8, 6.0)	0.199	12.4 (10.3, 14.5)	0.015
LSMM in T group		6.2 (4.5, 7.9)	0.629	12.0 (6.8, 17.2)	0.218
Patients diagnosed with LSMM after treatment with different therapies for 3 months					
LSMM-3m		5.1 (4.6, 5.6)	0.088	12.0 (9.9, 14.1)	0.002
I		4.1 (2.9, 5.4)	0.060	16.0	0.076
I+T		5.4 (4.8, 6.0)		12.0 (9.6, 14.3)	
T		6.1 (4.9, 7.2)		10.2 (5.4, 14.9)	
LSMM-3m in I group		4.1 (2.9, 5.4)	0.027	16.0	0.517
LSMM-3m in I+T group		5.4 (4.8, 6.0)	0.294	12.0 (9.6, 14.2)	<0.001
LSMM-3m in T group		6.1 (4.9, 7.2)	0.974	10.2 (5.4, 14.9)	0.190
Reduced ΔSMI with different therapies.					
Reduced ΔSMI		5.5 (5.1, 5.8)	0.297	13.8 (11.8, 15.8)	0.004
I		4.1 (3.2, 5.1)	0.487	16.0 (9.7, 22.3)	0.275
I+T		5.6 (5.1, 6.0)		12.0 (10.9, 15.2)	
T		6.0 (5.0, 7.0)		15.1 (9.4, 20.2)	
reduced ΔSMI in I group		4.1 (3.2, 5.1)	0.443	16.0 (9.7, 22.3)	0.821
reduced ΔSMI in I+T group		5.6 (5.1, 6.0)	0.542	13.0 (10.9, 15.2)	0.002
reduced ΔSMI in T group		6.0 (5.0, 7.0)	0.353	15.1 (9.4, 20.8)	0.887
Progressing LSMM with different therapies.					
progressing LSMM		4.9 (3.9, 5.8)	0.007	9.8 (7.9, 11.8)	<0.001
I		5.0 (4.0, 6.0)	0.643	10.8 (2.9, 18.7)	0.215
I+T		5.8 (5.5, 6.1)		9.7 (8.2, 11.1)	
T		6.1 (4.9, 7.2)		15.6 (4.0, 27.3)	
progressing LSMM in I group		4.2 (2.6, 5.9)	0.095	10.8 (2.9, 18.7)	0.107
progressing LSMM in I+T group		4.2 (2.9, 5.5)	0.013	9.7 (8.2, 11.1)	<0.001
progressing LSMM in T group		6.1 (3.7, 8.4)	0.876	15.6 (4.0, 27.3)	0.574

PFS, Progression-Free Survival; OS, Overall Survival; CI, Confidence Interval; TACE, Transarterial Chemoembolization; PVTT, Portal Vein Tumor Thrombosis; AFP, Alpha-Fetoprotein; BMI, Body Mass Index; ECOG-PS, Eastern Cooperative Oncology Group Performance Status; BCLC, Barcelona Clinic Liver Cancer; I, Immunotherapy; I+T, Targeted Therapy Combined with Immunotherapy; T, Targeted Therapy; LSMM, Low Skeletal Muscle Mass; ΔSMI, Change in Skeletal Muscle Index.

At the same time, we analyzed the relationship between the decrease in SMI after 3 months of treatment, progressive LSMM, and survival rates. Notably, compared to patients without a decrease in SMI, those with a decrease in SMI had a shorter OS (median, 13.8 *vs* 17.6, P=0.004), and this difference was also observed in the I+T group (median, 13.0 *vs* 17.0, P=0.002). However, a decrease in SMI was not related to PFS. In contrast, patients with progressive LSMM exhibited shorter PFS (median, 4.9 *vs* 5.7, P=0.007) and OS (median, 9.8 *vs* 16.5, P<0.001) compared to those without progressive LSMM. Similar differences were observed in the I+T group (PFS: median, 4.2 *vs*. 5.8, P=0.013;OS: median, 9.7 *vs* 16.1, P<0.001). (**Figure 4**). Additionally, age ≥ 60 years (P=0.008), distant metastasis (P=0.018) were associated with a shorter PFS. Pre-TACE treatment (P=0.010), distant metastasis (P=0.009), PVTT (P<0.001), and advanced BCLC staging (P=0.044) were associated with a shorter OS.

After performing multivariate COX regression analysis to control for confounding factors, as shown in **Table 8**, the presence of progressive LSMM was an independent risk factor for shorter OS and PFS (OS, HR = 1.533 [95% CI: 1.191-1.973], P<0.001; PFS, HR=1.321[95% CI:1.085-1.608], P=0.005). Additionally, age ≥60 years (HR = 1.257 [95% CI: 1.060-1.402], P=0.009) and distant metastasis (HR = 1.185 [95% CI: 1.001-1.402], P=0.049) were also independent risk factors for a lower PFS.

**Table 8:** Multifactorial COX regression analysis of factors associated with PFS and OS in all patients.

Variables	OS		PFS	
	HR (95%CI)	P	HR (95%CI)	P
Age≥60	/	/	1.257 (1.060-1.492)	0.009
Metastasis	1.039 (0.775-1.394)	0.797	1.185 (1.001-1.402)	0.049
LSMM	1.304 (0.980-1.735)	0.069	/	/
LSMM-3m	1.008 (0.753-1.348)	0.958	/	/
PVTT	1.285 (0.951-1.738)	0.103	/	/
Reduced ΔSMI	1.164 (0.911-1.488)	0.224	/	/
Progressing- LSMM	1.533 (1.191-1.973)	<0.001	1.321 (1.085-1.608)	0.005
Pre-TACE	0.811 (0.64-1.028)	0.083	/	/
BCLC-B	Ref.	Ref.	/	/
BCLC-C	1.169 (0.882-1.549)	0.277	/	/
BCLC-D	1.157 (0.715-1.87)	0.553	/	/

COX, Cox Proportional Hazards Model; PFS, Progression-Free Survival; OS, Overall Survival; HR, Hazard Ratio; CI, Confidence Interval; P, P-value; LSMM, Low Skeletal Muscle Mass; PVTT, Portal Vein Tumor Thrombosis; ΔSMI, Change in Skeletal Muscle Index; TACE, Transarterial Chemoembolization; BCLC, Barcelona Clinic Liver Cancer; Ref., Reference.

### 3.6 Analysis of Child-Pugh A patients

This study included 448 Child-Pugh A patients (48 in the I group, 347 in the I+T group, and 53 in the T group). Baseline characteristics are presented in **Supplementary Table 2**. The relationship between low muscle mass, changes in skeletal muscle mass, treatment regimens, and treatment response in Child-Pugh A patients was consistent with the overall population. However, unlike the overall cohort, baseline or post-treatment LSMM diagnosis did not significantly affect OS in Child-Pugh A patients.

#### 3.6.1 LSMM and skeletal muscle mass changes

In Child-Pugh A patients, baseline SMI did not differ significantly among the three groups, and baseline LSMM patients showed no significant difference in muscle loss(**Supplementary Table 3**). However, among baseline NLSMM patients, those in the I group had less muscle loss than those in the T group (P=0.018)(**Supplementary Figure 1**). As shown in **Supplementary Table 4**, muscle loss was significantly lower in LSMM patients than in NLSMM patients (P < 0.001), with the I+T group (P<0.001) and T group (P=0.008) showing similar trends.

#### 3.6.2 LSMM and treatment response

In Child-Pugh A patients, the objective response rate (ORR) was significantly associated with post-treatment LSMM diagnosis (P=0.013) (**Supplementary Table 5**). Patients who achieved ORR with combination therapy exhibited less muscle loss during treatment (P = 0.011).

#### 3.6.3 LSMM, SMI changes, and survival outcomes

Unlike the overall cohort, baseline and post-treatment LSMM diagnosis did not significantly impact OS in Child-Pugh A patients. However, The relationship between post-treatment SMI reduction, progressive LSMM, and survival rates in Child-Pugh A patients was consistent with the overall population. Patients with reduced SMI had shorter OS (median, 12.4 *vs*. 17.6 months, P=0.014; Group I+T: 11.7 *vs*. 12.3 months, P=0.001). Progressive LSMM was associated with shorter PFS (median, 4.8 *vs*. 5.9 months, P=0.016; Group I+T: 4.0 *vs*. 5.8 months, P=0.039) and OS (median, 9.5 *vs*. 16.5 months, P<0.001; Group I+T: 8.9 *vs*. 16.5 months, P<0.001) (**Supplementary Table 6**).

#### 3.6.4 Multivariate COX regression analysis of PFS and OS in Child-Pugh A patients

As shown in **Supplementary Table 6**, progressive LSMM was an independent risk factor for both shorter OS (HR = 1.581, 95% CI: 1.167–2.140, P = 0.003) and PFS (HR = 1.361, 95% CI: 1.066–1.738, P = 0.013). Additionally, Age ≥ 60 years was an independent risk factor for shorter PFS (HR = 1.272, 95% CI: 1.027–1.577, P = 0.028).PVTT was an independent risk factor for shorter OS (HR = 1.426, 95% CI: 1.086–1.874, P = 0.011).

These findings align with the overall cohort, indicating that in Child-Pugh A patients, baseline muscle status—especially L3-SMI and SMI changes—are closely linked to treatment response and prognosis (PFS and OS).

### 3.7 Treatment-related severe adverse events

As presented in **Table 9**, among the 714 patients receiving systemic treatment for HCC, the most common treatment-related SAEs were ascites (19.2%), fatigue (8.7%), and loss of appetite (4.2%), with no significant differences among treatment groups. Additionally, patients with LSMM exhibited a higher overall incidence of SAEs, with fatigue (12.8% *vs*. 6.1%, P = 0.002) and loss of appetite (6.6% *vs*. 2.7%, P = 0.012) being significantly more common in the LSMM group (**Table 10**). This suggests that reduced muscle mass may impact treatment tolerance and quality of life. Other SAEs, including gastrointestinal bleeding, infection, jaundice, liver dysfunction, hypothyroidism, and rash, showed no statistically significant differences across treatment groups or between LSMM and NLSMM patients. These findings indicate that LSMM may increase the risk of treatment-related adverse events, highlighting the need for close monitoring and supportive interventions in these patients.

**Table 9:** Treatment-related severe adverse events (SAE) in different treatment groups.

Variables	Total (n = 714)	I (n = 85)	I+T (n = 545)	T (n = 84)	*P*
Gastrointestinal bleeding, n(%)	17 (2.38)	0 (0.00)	17 (3.12)	0 (0.00)	0.075
Infection, n(%)	25 (3.50)	2 (2.35)	23 (4.22)	0 (0.00)	0.125
Ascites, n(%)	137 (19.19)	13 (15.29)	112 (20.55)	12 (14.29)	0.248
Hepatic encephalopathy, n(%)	10 (1.40)	1 (1.18)	9 (1.65)	0 (0.00)	0.853
Jaundice, n(%)	24 (3.36)	4 (4.71)	19 (3.49)	1 (1.19)	0.450
Liver dysfuction, n(%)	25 (3.50)	4 (4.71)	20 (3.67)	1 (1.19)	0.441
Thrombocytopenia, n(%)	5 (0.70)	0 (0.00)	5 (0.92)	0 (0.00)	1.000
Fatigue, n(%)	62 (8.68)	9 (10.59)	45 (8.26)	8 (9.52)	0.745
Loss Of Appetite, n(%)	30 (4.20)	3 (3.53)	27 (4.95)	0 (0.00)	0.083
Hypothyroidism, n(%)	5 (0.70)	1 (1.18)	4 (0.73)	0 (0.00)	0.742
Diarrhea, n(%)	2 (0.28)	1 (1.18)	1 (0.18)	0 (0.00)	0.418
Myocardial Injury, n(%)	1 (0.14)	1 (1.18)	0 (0.00)	0 (0.00)	0.237
Hepatorenal Syndrome, n(%)	3 (0.42)	1 (1.18)	2 (0.37)	0 (0.00)	0.556
Rash, n(%)	4 (0.56)	1 (1.18)	2 (0.37)	1 (1.19)	0.239
Severe Neuropenia, n(%)	2 (0.28)	0 (0.00)	2 (0.37)	0 (0.00)	1.000

**Table 10:** Treatment-related serious adverse events in LSMM and NLSMM groups.

Variables	Total (n = 714)	LSMM (n = 273)	NLSMM (n = 441)	*P*
Gastrointestinal bleeding, n(%)	17 (2.38)	10 (3.66)	7 (1.59)	0.077
Infection, n(%)	25 (3.50)	8 (2.93)	17 (3.85)	0.514
Ascites, n(%)	137 (19.19)	58 (21.25)	79 (17.91)	0.272
Hepatic encephalopathy, n(%)	10 (1.40)	5 (1.83)	5 (1.13)	0.658
Jaundice, n(%)	24 (3.36)	7 (2.56)	17 (3.85)	0.352
Liver dysfuction, n(%)	25 (3.50)	8 (2.93)	17 (3.85)	0.514
Thrombocytopenia, n(%)	5 (0.70)	3 (1.10)	2 (0.45)	0.587
Fatigue, n(%)	62 (8.68)	35 (12.82)	27 (6.12)	0.002
Loss Of Appetite, n(%)	30 (4.20)	18 (6.59)	12 (2.72)	0.012
Hypothyroidism, n(%)	5 (0.70)	2 (0.73)	3 (0.68)	1.000
Diarrhea, n(%)	2 (0.28)	1 (0.37)	1 (0.23)	1.000
Myocardial Injury, n(%)	1 (0.14)	0 (0.00)	1 (0.23)	1.000
Hepatorenal Syndrome, n(%)	3 (0.42)	0 (0.00)	3 (0.68)	0.441
Rash, n(%)	4 (0.56)	3 (1.10)	1 (0.23)	0.317
Severe Neuropenia, n(%)	2 (0.28)	0 (0.00)	2 (0.45)	0.527

SAE, serious adverse event; LSMM, low skeletal muscle mass; NLSMM, non-low skeletal muscle mass; I, immunotherapy; I+T, immunotherapy plus targeted therapy; T, targeted therapy; P, P-value were used in the analysis.

### 3.8 Multivariate COX regression analysis of factors related to PFS and OS in different treatment groups

There were no significant differences in median survival and progression-free survival among the three treatment groups. Subgroup analysis of the aforementioned factors, as shown in **Table 11**, revealed that the univariate analysis results for PFS and OS in the I+T group were similar to those of the overall patient population. Multivariate analysis showed that progressive LSMM was an independent risk factor for shorter PFS and OS (PFS, HR = 1.338 [95% CI: 1.066-1.680], P=0.012; OS, HR = 1.839 [95% CI: 1.435-2.351], P<0.001); additionally, prior TACE treatment was an independent protective factor for OS (HR = 0.725 [95% CI: 0.562-0.936], P=0.014). Age ≥ 60 years was an independent risk factor for progression (HR = 1.236 [95% CI: 1.014-1.505], P=0.036).

**Table 11:** Multifactorial COX regression analysis of factors associated with PFS and OS in three groups.

Group	Variables	OS		PFS	
		HR (95%CI)	P	HR (95%CI)	P
**I+T**	Age≥60	/	/	1.236 (1.014-1.505)	0.036
	Metastasis	1.039 (0.775-1.394)	0.797	1.196 (0.986-1.451)	0.069
	LSMM	1.369 (0.985-1.902)	0.062	/	/
	LSMM-3m	1.016 (0.727-1.420)	0.927	/	/
	PVTT	1.228 (0.874-1.724)	0.236	/	/
	Progressing- LSMM	1.839 (1.435-2.357)	<0.001	1.338 (1.066-1.680)	0.012
	Pre-TACE	0.725 (0.562-0.936)	0.014	/	/
**I**	Metastasis	1.262 (0.538-2.960)	0.592	/	/
	LSMM-3m	/	/	1.831 (1.105-3.033)	0.019
	PVTT	4.286 (1.546-7.885)	0.005	2.288 (1.251-4.185)	0.007
	Progressing LSMM	/	/	/	/
	Pre-TACE	3.327 (1.570-7.470)	0.002	/	/
**T**	AFP ≥ 400 ng/ml	1.978 (1.095-3.571)	0.024	/	/

COX, Cox Proportional Hazards Model; PFS, Progression-Free Survival; OS, Overall Survival; HR, Hazard Ratio; CI, Confidence Interval; P, P-value; I, Immunotherapy; I+T, Targeted Therapy Combined with Immunotherapy; T, Targeted Therapy; LSMM, Low Skeletal Muscle Mass; PVTT, Portal Vein Tumor Thrombosis; TACE, Transarterial Chemoembolization; AFP, Alpha-Fetoprotein.

In the univariate analysis of Group I, distant metastasis, PVTT, and prior surgical treatment were significantly associated with OS. Subsequent multivariate analysis identified the presence of PVTT (HR = 4.286 [95% CI: 1.546-7.885], P=0.005) and prior surgical treatment (HR = 3.327 [95% CI: 1.570-7.470], P=0.002) as independent risk factors for OS in HCC patients treated with Group I. The independent risk factors for PFS were PVTT (HR = 1.831 [95% CI: 1.105-3.033], P=0.019) and the diagnosis of LSMM after 3 months of treatment (HR = 2.288 [95% CI: 1.251-4.185], P=0.007).

Among HCC patients treated with Group T, only AFP >400ng/ml was an independent risk factor for OS (HR = 1.978 [95% CI: 1.095-3.571], P=0.024).

## 4 Discussion

This study aimed to assess the impact of I, I+T, and T treatments on skeletal muscle mass changes in patients with intermediate to advanced hepatocellular carcinoma and to explore the influence of LSMM and progressive LSMM on treatment outcomes and prognosis across different treatment groups. The findings revealed no difference in muscle mass loss among LSMM patients receiving I, I+T, and T treatments; however, T treatment appeared to promote muscle loss. Patients with greater muscle loss experienced poorer treatment outcomes, and those with baseline LSMM, post-treatment LSMM, and progressive LSMM had significantly worse prognoses, especially in the I+T group.

In intermediate to advanced HCC patients, specific demographic characteristics and treatment statuses are potential risk factors for LSMM. The first-line treatment was negatively correlated with an increased likelihood of baseline muscle wasting, suggesting that the incidence of muscle wasting is lower in first-line treatment, and patients with muscle wasting are often diagnosed when receiving non-first-line treatments. It has been reported that in cancer patients, the causes of skeletal muscle depletion include reduced physical activity and malnutrition due to disease progression and adverse effects of treatment, as well as increased expression of inflammatory cytokines (14). Regardless of tumor progression, patients receiving systemic treatment lose skeletal muscle (15). There was no significant correlation between different treatment regimens and post-treatment LSMM, and baseline LSMM diagnosis and the presence of progressive muscle wasting were significant correlates of post-treatment muscle wasting. This emphasizes the impact of muscle condition at baseline on the development of LSMM during subsequent treatments and the increased risk of muscle wasting due to the progression of LSMM. This study found that elderly individuals (aged ≥60 years) and males were significantly correlated with baseline LSMM, consistent with previous research findings (16, 17), and possibly related to the decrease in testosterone associated with aging, a hormone that promotes skeletal muscle growth. On the other hand, females are generally more inclined to store a significant amount of fat from fat reserves and generate energy, rather than from skeletal muscle reserves (18), which may make them more resistant to muscle wasting compared to males.

Our observations indicate that there was no significant difference in muscle wasting among patients with baseline LSMM treated with the three regimens, while NLSMM patients in the T group experienced more muscle loss than those in the I group. Comparing muscle loss during treatment between LSMM and NLSMM patients within each group revealed that in the I+T and T groups, LSMM patients lost less muscle mass than NLSMM patients, while the I regimen had no significant impact on muscle changes in patients with different baseline nutritional statuses. This suggests that T may have a promoting effect on muscle loss in NLSMM patients, potentially due to dose toxicity and direct mechanisms that induce muscle wasting, such as alterations in the PI3K/AKT-mTOR signaling pathway. mTOR (mammalian target of rapamycin) is a key regulator of muscle protein synthesis, with its primary complex, mTORC1, controlling protein synthesis, cell growth, metabolism, and autophagy by phosphorylating downstream targets. Inhibiting mTORC1 enhances autophagy and mitophagy, facilitating the clearance of damaged mitochondria and maintaining muscle homeostasis, while mTORC1 also negatively regulates autophagy to preserve protein synthesis balance (19, 20). Activation of the PI3K/AKT/mTOR pathway promotes muscle growth (21), whereas T therapy may suppress this pathway, impairing muscle protein synthesis and exacerbating sarcopenia (22). Additionally, TKIs (tyrosine kinase inhibitors) may promote protein degradation by reducing phosphorylation of mTOR downstream targets, such as p70S6K and 4E-BP1, further contributing to muscle loss (23). VEGF (vascular endothelial growth factor) plays a crucial role in maintaining skeletal muscle blood flow and neovascularization. VEGF inhibitors (such as bevacizumab) may reduce muscle perfusion, leading to diminished nutrient supply and increased muscle wasting. However, in this study, no significant increase in muscle loss was observed in the I+T group due to VEGF inhibition. This may be attributed to the overall physiological effects of combination therapy. For instance, ICIs (immune checkpoint inhibitors) may reduce systemic inflammation (e.g., IL-6, TNF-α), improving metabolic homeostasis and partially counteracting TKI-induced muscle loss. These results indicate that LSMM should be considered a factor in the decision-making for protein kinase inhibitor TKI treatment in HCC patients.

We found that the diagnosis of muscle wasting at baseline was not significantly associated with the effectiveness of tumor treatment both in the entire cohort and in Child-Pugh A patients, while achieving an ORR was related to the diagnosis of LSMM after three months of treatment. A significant decline in SMI was observed in patients treated with I (-2.03), I+T (-1.34), and T (-1.83), and patients with greater muscle loss had poorer treatment outcomes, a phenomenon particularly evident in the I+T group. This is consistent with previous research findings (13, 15).

Current studies on the prognostic impact of LSMM in HCC patients receiving targeted therapy, immunotherapy, and combined therapy show some controversy. Some studies suggest that LSMM is associated with PFS and OS in advanced HCC patients treated with TKIs, including sorafenib and lenvatinib Sun et al. (24–26) believe that LSMM does not determine PFS and OS in advanced HCC patients treated with lenvatinib combined with PD-1 inhibitors (27). Matsumoto et al. found no significant correlation between the presence of LSMM and PFS in HCC patients treated with atezolizumab/becatecarin (16), while Ourak et al. showed that LSMM patients had significantly shorter PFS and OS than NLSMM patients (28). Liu, M et al. indicated that baseline LSMM was not related to PFS and OS in HCC patients treated with ICIs (13). The subgroup analysis in our study provides stronger evidence for the impact of LSMM on OS and PFS in patients receiving the same treatment. The results of this study suggest that in the entire cohort, LSMM is significantly associated with survival time in intermediate to advanced HCC patients, both at baseline and post-treatment, and progressive LSMM is an independent risk factor for shorter OS and PFS, especially in the I+T treatment group. In contrast, baseline LSMM in patients receiving TKI treatment was not related to PFS and OS, which is inconsistent with previous study results (13, 16, 24–26). This may be due to smaller patient sample sizes, selection differences, and different combinations of treatment drugs. In this study, baseline LSMM in patients treated with ICI was not related to PFS and OS, which is consistent with previous research findings (13, 29). We also observed that a diagnosis of LSMM post-treatment in HCC patients treated with ICI was significantly correlated with PFS, supplementing the conclusions of Akce et al. (29).

In the entire cohort, both baseline and post-treatment LSMM were significantly associated with survival in intermediate to advanced HCC. However, in Child-Pugh A patients, LSMM had no significant impact on survival, while progressive LSMM correlated with poorer PFS and OS. This suggests that patients with better liver function tolerate muscle loss better, whereas LSMM has a greater prognostic impact in those with impaired liver function. Future research and interventions should consider liver function stratification, with a focus on dynamic LSMM monitoring to identify high-risk patients and optimize nutritional and rehabilitation strategies.

The adverse impact of LSMM on the effectiveness and prognosis of HCC treatment may be related to the tumor microenvironment (inflammation and immunity) and cytokine activity. It has been reported that skeletal muscle is an organ with immunomodulatory properties, regulating immune function through various soluble factors, cell surface molecules, or cell-to-cell interactions (30, 31). It can produce myokines such as IL-15 to mitigate the harmful effects of pro-inflammatory adipokines, contributing to the suppressive effects on the tumor microenvironment (30, 32). The homeostasis of skeletal muscle is to some extent the cause of healthy immune function, and when muscle atrophy occurs, it reduces the skeletal muscle cell signaling required for immune modulation and maintenance, leading to systemic inflammation and immune disorders (31, 33). On the other hand, skeletal muscle cells can express major histocompatibility complex molecules, delivering antigens to T cells. The reduction in muscle mass may mediate tumor cell immune tolerance to ICIs by affecting T cell function (31). Skeletal muscle wasting can also lead to a decrease in myoglobin levels, which may result in poor responses to immunotherapy (30, 32). In summary, LSMM may affect immune modulation, leading to poorer outcomes in HCC patients.

This study found a higher incidence of SAEs in LSMM patients, particularly fatigue and appetite loss, likely due to metabolic dysfunction, systemic inflammation, and immune dysregulation. SAE rates did not differ significantly between treatment groups, suggesting muscle status may better predict SAE risk than treatment regimen. Increased SAEs in LSMM patients may reduce treatment tolerance, leading to dose adjustments or discontinuation, ultimately impacting survival. Close monitoring, along with nutritional and exercise interventions, may help improve patient outcomes.

Additionally, multivariate analysis also revealed that the elderly (age ≥60 years) and extrahepatic metastasis are independent risk factors for PFS in HCC patients receiving systemic treatment. In subgroup analysis, PVTT and prior surgical treatment were independent risk factors for OS in Group I, while PVTT and post-treatment LSMM were independent risk factors for PFS. In the I+T group, the elderly (age ≥60 years) were independent risk factors for progression, and prior TACE treatment was an independent protective factor for OS. In Group T, only AFP >400ng/ml was an independent risk factor for OS. These results provide specific factors affecting prognosis in different treatment groups.

These results suggest that there is no difference in the impact on muscle mass due to LSMM across different treatment groups, while T treatment has a direct loss effect on muscle mass, which should be an important consideration in treatment decision-making and prognosis assessment. At the same time, they emphasize the significant role of LSMM and progressive LSMM in predicting the prognosis of intermediate to advanced HCC patients, especially in the I+T treatment group, and suggest that clinicians should pay attention to the monitoring and management of LSMM in clinical practice. Exercise and nutritional interventions can enhance the metabolism and function of skeletal muscle (34). Regular resistance training and aerobic exercise are widely recognized as effective strategies for improving muscle mass and function. Supplementing with branched-chain amino acids (BCAAs) can help correct hypoalbuminemia, reduce skeletal muscle fat accumulation, maintain muscle mass, and improve glucose sensitivity, thereby preventing sarcopenia in patients with chronic liver disease (35). Additionally, Levocarnitine supplementation has shown potential benefits in improving skeletal muscle mass in HCC patients receiving lenvatinib treatment (36). Integrating multiple muscle-preserving interventions into patient treatment plans—such as personalized resistance training, Levocarnitine, protein, and amino acid supplementation—may help restore muscle function and support immune system health. Furthermore, chemotherapeutic agents and immunosuppressants may cause muscle loss; dose adjustments or alternative therapies could help mitigate this while maintaining treatment efficacy. However, these strategies require strict medical supervision.

This study is a single-center retrospective cohort study and has certain limitations. The study population has a low proportion of females, and the number of patients in the immunotherapy and targeted therapy groups is less than in the combined group. Patients who did not undergo CT scans at our hospital before treatment were not included in this study, which may cause selection bias. Secondly, the specific drugs and dosages of different treatment regimens varied from patient to patient, causing bias. Moreover, after discontinuing the corresponding targeted drugs, immunotherapy drugs, or combined medications, various other anticancer therapies may be used subsequently, which could bias the clinical outcomes of the patients. Finally, the error between manually outlining muscle area and the actual situation cannot be completely eliminated; future studies should validate our results in a broader population and with additional basic research.

## Data Availability

The original contributions presented in the study are included in the article/**Supplementary Material**. Further inquiries can be directed to the corresponding author.
