# Supplementary material for: The relationship between systemic therapies and low skeletal muscle mass in patients with intermediate and advanced hepatocellular carcinoma
Source: Front Immunol. 2025 Mar 5;16:1557839. doi: 10.3389/fimmu.2025.1557839 (PMC11919905; doi:10.3389/fimmu.2025.1557839)
Supplement: Supplementary file 1 [file DataSheet1.docx]

Supplementary Material

# Supplementary Table 1. Baseline characteristics of complete response(CR) patients.

| Variables | Total (n = 53) | I (n = 6) | I+T (n = 42) | T (n = 5) | *P* |
| --- | --- | --- | --- | --- | --- |
|  |  |  |  |  |  |
| Age, Mean ± SD | 61.19 ± 8.84 | 58.67 ± 10.13 | 61.14 ± 8.37 | 64.60 ± 12.07 | 0.549 |
| Gender(Male), n(%) | 43 (81.13) | 3 (50.00) | 35 (83.33) | 5 (100.00) | 0.113 |
| BMI, Mean ± SD | 24.24 ± 3.80 | 22.43 ± 2.16 | 24.34 ± 3.64 | 25.62 ± 6.24 | 0.367 |
| L3-SMI, Mean ± SD | 44.41 ± 6.91 | 45.70 ± 7.87 | 43.99 ± 6.86 | 46.45 ± 7.24 | 0.679 |
| LSMM, n(%) | 18 (33.96) | 1 (16.67) | 15 (35.71) | 2 (40.00) | 0.763 |
| HBV, n(%) | 53 (100) | 6 (100) | 42 (100) | 5 (100) | 1.000 |
| HCV, n(%) | 9 (16.98) | 0 (0.00) | 9 (21.43) | 0 (0.00) | 0.524 |
| Child-Pugh, n(%) |  |  |  |  | 0.146 |
| A | 28 (52.83) | 3 (50.00) | 24 (57.14) | 1 (20.00) |  |
| B | 22 (41.51) | 2 (33.33) | 17 (40.48) | 3 (60.00) |  |
| C | 3 (5.66) | 1 (16.67) | 1 (2.38) | 1 (20.00) |  |
| BCLC, n(%) |  |  |  |  | 0.065 |
| B | 18 (33.96) | 0 (0.00) | 17 (40.48) | 1 (20.00) |  |
| C | 32 (60.38) | 5 (83.33) | 24 (57.14) | 3 (60.00) |  |
| D | 3 (5.66) | 1 (16.67) | 1 (2.38) | 1 (20.00) |  |
| ECOG-PS, n(%) |  |  |  |  | 1.000 |
| 0 | 2 (3.77) | 0 (0.00) | 2 (4.76) | 0 (0.00) |  |
| 1 | 49 (92.45) | 6 (100.00) | 38 (90.48) | 5 (100.00) |  |
| 2 | 2 (3.77) | 0 (0.00) | 2 (4.76) | 0 (0.00) |  |
| PVTT, n(%) | 4 (7.55) | 0 (0.00) | 4 (9.52) | 0 (0.00) | 1.000 |
| Metastasis, n(%) | 14 (26.42) | 2 (33.33) | 12 (28.57) | 0 (0.00) | 0.530 |
| Cirrhosis, n(%) | 48 (90.57) | 6 (100.00) | 37 (88.10) | 5 (100.00) | 1.000 |
| First-line, n(%) | 34 (64.15) | 5 (83.33) | 27 (64.29) | 2 (40.00) | 0.384 |
| Line, n(%) |  |  |  |  | 0.618 |
| 1 | 34 (64.15) | 5 (83.33) | 27 (64.29) | 2 (40.00) |  |
| 2 | 12 (22.64) | 1 (16.67) | 9 (21.43) | 2 (40.00) |  |
| 3 | 7 (13.21) | 0 (0.00) | 6 (14.29) | 1 (20.00) |  |
| Pre-Surgery, n(%) | 20 (37.74) | 2 (33.33) | 18 (42.86) | 0 (0.00) | 0.224 |
| Pre-TACE, n(%) | 48 (90.57) | 3 (50.00) | 40 (95.24) | 5 (100.00) | 0.014 |
| Pre-Systematic treatment, n(%) | 19 (35.85) | 1 (16.67) | 15 (35.71) | 3 (60.00) | 0.384 |
| AFP≥ 400(ng/mL), n(%) | 17 (32.08) | 3 (50.00) | 12 (28.57) | 2 (40.00) | 0.490 |

Abbreviations: I (Immunotherapy); I+T (Targeted Therapy Combined with Immunotherapy); T (Targeted Therapy);P (P-value);BMI (Body Mass Index); L3-SMI (Third Lumbar Skeletal Muscle Index); LSMM (Low Skeletal Muscle Mass); HBV (Hepatitis B Virus); HCV (Hepatitis C Virus); BCLC (Barcelona Clinic Liver Cancer); ECOG-PS (Eastern Cooperative Oncology Group Performance Status); PVTT (Portal Vein Tumor Thrombosis); TACE (Transarterial Chemoembolization); AFP (Alpha-Fetoprotein); CR (Complete Response). (Multiple group comparisons: ANOVA for normal data, Kruskal-Wallis test for non-normal data.)

# Supplementary Table 2. Baseline characteristics of Child-Pugh A patients.

| Variables | Total (n = 448) | I(n = 48) | I+T (n = 347) | T (n = 53) | *P* |
| --- | --- | --- | --- | --- | --- |
| Age, Mean ± SD | 58.56 ± 10.09 | 62.62 ± 10.23 | 57.98 ± 9.80 | 58.66 ± 11.14 | 0.011 |
| Gender(male), n(%) | 388 (86.61) | 302 (87.03) | 40 (83.33) | 46 (86.79) | 0.779 |
| BMI, Mean ± SD | 24.07 ± 3.66 | 24.16 ± 3.49 | 24.07 ± 3.68 | 23.98 ± 3.69 | 0.972 |
| L3-SMI(cm2/m2)，Mean ± SD | 44.35 ± 8.50 | 42.73 ± 7.83 | 44.48 ± 8.71 | 44.91 ± 7.60 | 0.356 |
| LSMM, n(%) | 168 (37.50) | 131 (37.75) | 18 (37.50) | 19 (35.85) | 0.965 |
| HBV,n(%) | 422 (94.20) | 45 (93.75) | 331 (95.39) | 46 (86.79) | 0.045 |
| HCV,n(%) | 70 (15.62) | 8 (16.67) | 52 (14.99) | 10 (18.87) | 0.752 |
| BCLC, n(%) |  |  |  |  | 0.580 |
| B | 112 (25.00) | 83 (23.92) | 13 (27.08) | 16 (30.19) |  |
| C | 336 (75.00) | 264 (76.08) | 35 (72.92) | 37 (69.81) |  |
| ECOG-PS, n(%) |  |  |  |  | 0.991 |
| 0 | 14 (3.12) | 11 (3.17) | 1 (2.08) | 2 (3.77) |  |
| 1 | 414 (92.41) | 320 (92.22) | 45 (93.75) | 49 (92.45) |  |
| 2 | 20 (4.46) | 16 (4.61) | 2 (4.17) | 2 (3.77) |  |
| PVTT,n(%) | 125 (27.90) | 7 (14.58) | 101 (29.11) | 17 (32.08) | 0.095 |
| Metastasis,n(%) | 206 (45.98) | 15 (31.25) | 166 (47.84) | 25 (47.17) | 0.085 |
| Cirrhosis, n(%) | 417 (93.08) | 45 (93.75) | 324 (93.37) | 48 (90.57) | 0.680 |
| First-line,n(%) | 280 (62.50) | 20 (41.67) | 223 (64.27) | 37 (69.81) | 0.005 |
| Line, n(%) |  |  |  |  | 0.015 |
| 1 | 280 (62.50) | 20 (41.67) | 223 (64.27) | 37 (69.81) |  |
| 2 | 98 (21.88) | 14 (29.17) | 73 (21.04) | 11 (20.75) |  |
| 3 | 70 (15.62) | 14 (29.17) | 51 (14.70) | 5 (9.43) |  |
| Pre-Surgery,n(%) | 69 (15.40) | 15 (31.25) | 47 (13.54) | 7 (13.21) | 0.006 |
| Pre-TACE,n(%) | 371 (82.81) | 39 (81.25) | 284 (81.84) | 48 (90.57) | 0.280 |
| Pre-Systematic theraphy,n(%) | 168 (37.50) | 28 (58.33) | 124 (35.73) | 16 (30.19) | 0.005 |
| AFP≥400(ng/ml),n(%) | 174 (38.84) | 17 (35.42) | 144 (41.50) | 13 (24.53) | 0.054 |
| Tumor response in 3 months, n(%) |  |  |  |  | 0.182 |
| CR | 28 (6.25) | 3 (6.25) | 24 (6.92) | 1 (1.89) |  |
| PD | 156 (34.82) | 18 (37.50) | 122 (35.16) | 16 (30.19) |  |
| PR | 100 (22.32) | 5 (10.42) | 78 (22.48) | 17 (32.08) |  |
| SD | 164 (36.61) | 22 (45.83) | 123 (35.45) | 19 (35.85) |  |
| ORR, n(%) | 128 (28.57) | 8 (16.67) | 102 (29.39) | 18 (33.96) | 0.122 |
| DCR, n(%) | 292 (65.18) | 30 (62.50) | 225 (64.84) | 37 (69.81) | 0.715 |

Abbreviations: I (Immunotherapy); I+T (Targeted Therapy Combined with Immunotherapy); T (Targeted Therapy);P (P-value);BMI (Body Mass Index); L3-SMI (Third Lumbar Skeletal Muscle Index); LSMM (Low Skeletal Muscle Mass); HBV (Hepatitis B Virus); HCV (Hepatitis C Virus); BCLC (Barcelona Clinic Liver Cancer); ECOG-PS (Eastern Cooperative Oncology Group Performance Status); PVTT (Portal Vein Tumor Thrombosis); TACE (Transarterial Chemoembolization); AFP (Alpha-Fetoprotein); CR (Complete Response); PD (Progressive Disease); PR (Partial Response); SD (Stable Disease); ORR (Objective Response Rate); DCR (Disease Control Rate). (Multiple group comparisons: ANOVA for normal data, Kruskal-Wallis test for non-normal data.）

# Supplementary Table 3. SMI and ∆SMI in Child-Pugh A subgroup patients across different groups.

| **Variables** | **I** | **I+T** | **T** | **Total** | **p** |
| --- | --- | --- | --- | --- | --- |
|  | **n=48** | **n=347** | **n=53** | **n=448** |  |
| SMI (cm2/m2) Mean ± SD | | | | | |
| baseline | 42.73±7.83 | 44.48±8.71 | 44.91±7.60 | 44.35±8.50 | 0.877 |
| at 3 months | 42.00±8.27 | 42.65±8.79 | 42.42±7.02 | 42.55±8.53 | 0.054 |
| ΔSMI (cm2/m2) Mean ± SD | | | | | |
| in all patients | -1.21±3.72 | -1.86±4.37 | -2.25±5.23 | -1.83±4.41 | 0.130 |
| in LSMM patients | 0.04±3.91 | -0.80±3.80 | 0.13±3.61 | -0.60±3.79 | 0.458 |
| in NLSMM patients | -1.19±3.99 | -2.47±4.52 | -3.96±5.82 | -2.51±4.67 | 0.058 |

Abbreviations:SMI (Skeletal Muscle Index); ∆SMI (Change in Skeletal Muscle Index); I (Immunotherapy); I+T (Targeted Therapy Combined with Immunotherapy); T (Targeted Therapy); P (P-value); SD (Standard Deviation); LSMM (Low Skeletal Muscle Mass).

1. **Supplementary Table 4. Impact of treatments on ∆SMI in Child-Pugh A patients by baseline muscle status (intra-group).**

| **ΔSMI (cm2/m2) Mean ± SD** | **LSMM** | | **Total** | **p** |
| --- | --- | --- | --- | --- |
|  | **Yes** | **No** |  |  |
| total | -0.60±3.79 | -2.51±4.67 | -1.80±4.45 | <0.001 |
| I | 0.04±3.91 | -1.19±4.00 | -0.73±3.96 | 0.305 |
| I+T | -0.80±3.80 | -2.47±4.52 | -1.84±4.33 | <0.001 |
| T | -0.13±3.61 | -4.00±5.82 | -2.49±5.47 | 0.008 |

Abbreviations:SMI (Skeletal Muscle Index); ∆SMI (Change in Skeletal Muscle Index); I (Immunotherapy); I+T (Targeted Therapy Combined with Immunotherapy); T (Targeted Therapy); P (P-value); SD (Standard Deviation); LSMM (Low Skeletal Muscle Mass).

1. **Supplementary Table 5. Relationship between muscle mass changes and treatment response in Child-Pugh A patients.**

| **Variables** | | **ORR-3months** | | | |
| --- | --- | --- | --- | --- | --- |
|  |  | **0** | **1** | **total** | **P** |
| SMI-baseline  (cm2/m2)Mean±SD | | 43.87±8.48 | 45.55±8.47 | 44.35±8.51 | 0.058 |
| SMI-3months  (cm2/m2)Mean±SD | | 41.87±8.47 | 44.25±8.48 | 42.56±8.53 | 0.008 |
| LSMM-baseline | | 129(40.3%) | 39(30.2%) | 168(37.5%) | 0.052 |
| LSMM-3months | | 164(51.3%) | 49(38.3%) | 213(47.5%) | 0.013 |
| Progressing LSMM | | 79(24.7%) | 22(17.2%) | 101(22.5%) | 0.086 |
| ΔSMI(cm2/m2)Mean±SD | | -1.99±4.51 | -1.30±4.30 | -1.80±4.45 | 0.136 |
| ΔSMI and treatment response in patients with and without baseline LSMM. | | | | | |
| LSMM patients | | -0.68±3.87 | -0.34±3.52 | -0.60±3.79 | 0.619 |
| NLSMM patients | | -2.88±4.69 | -1.72±4.55 | -2.51±4.67 | 0.053 |
| ∆SMI and treatment response across therapeutic regimens. | | | | | |
| I | -0.47±4.20 | | -2.02±2.23 | -0.73±3.96 | 0.319 |
| I+T | -2.22±4.48 | | -0.92±3.83 | -1.84±4.33 | 0.011 |
| T | -2.17±4.83 | | -3.12±6.64 | -2.49±5.47 | 0.551 |
| ΔSMI and treatment response in LSMM patients across groups. | | | | | |
| I | 0.04±3.91 | | / | 0.04±3.91 | / |
| I+T | -1.04±3.89 | | -0.13±3.49 | -0.80±3.80 | 0.227 |
| T | -1.04±3.89 | | -0.13±3.49 | -0.80±3.80 | 0.161 |
| ΔSMI and treatment response in NLSMM using across groups. | | | | | |
| I | -0.89±4.46 | | -2.02±2.23 | -1.19±3.99 | 0.502 |
| I+T | -2.98±4.67 | | -1.33±3.95 | -2.47±4.52 | 0.013 |
| T | -4.34±4.62 | | -3.40±7.36 | -3.96±5.82 | 0.650 |

Abbreviations:SMI (Skeletal Muscle Index); ∆SMI (Change in Skeletal Muscle Index); I (Immunotherapy); I+T (Targeted Therapy Combined with Immunotherapy); T (Targeted Therapy); P (P-value); SD (Standard Deviation); LSMM (Low Skeletal Muscle Mass); ORR (Objective Response Rate); NLSMM (Non-Low Skeletal Muscle Mass).

1. **Supplementary Table 6. Kaplan-Meier analysis of PFS and OS factors in Child-Pugh A patients.**

| Variables | | PFS | | OS | |
| --- | --- | --- | --- | --- | --- |
|  |  | Median time 95% CI (months) | P | Median time 95% CI (months) | P |
| Baseline | | | | | |
| Age≥60 | | 6.0(5.4,6.7) | 0.030 | 15.0(12.9,17.1) | 0.775 |
| Gender (Male) | | 4.8(3.3,6.3) | 0.758 | 15.4(13.5,17.2) | 0.587 |
| First line | | 5.7(5.3,6.3) | 0.205 | 16.0(14.1,18.0) | 0.684 |
| PreSurgery | | 5.8(4.2,7.3) | 0.396 | 14.9(12.1,17.7) | 0.662 |
| PreTACE | | 5.7(5.4,6.1) | 0.447 | 14.5(12.4,16.6) | 0.614 |
| Metastasis | | 5.1(4.4,5,9) | 0.039 | 12.2(9.8,14.5) | 0.045 |
| PVTT | | 5.4(4.8,6.0) | 0.275 | 10.3(8.8,11.7) | 0.004 |
| AFP≥400ng/ml | | 5.6(4.9,6.2) | 0.974 | 13.6(10.4,16.8) | 0.261 |
| Obesity(BMI≥24kg/m2) | | 5.7(5.2,6.0) | 0.179 | 16.1(13.5,18.6) | 0.830 |
| Underweight(BMI≤18.5kg/m2) | | 5.6(5.2,6.0) | 0.323 | 12.6(8.8,16.4) | 0.942 |
| ECOG-PS | 0 | 8.9(6.3,11.5) | 0.249 | 24.1(16.2,32.1) | 0.134 |
|  | 1 | 5.6(5.2,6.0) |  | 14.9(12.7,17.2) |  |
|  | 2 | 4.8(2.9,6.8) |  | 13.2(10.1,15.5) |  |
| BCLC | B | 5.8(5.2,6.5) | 0.868 | 16.1(11.8,20.4) | 0.110 |
|  | C | 5.6(5.1,6.1) |  | 14.0(11.6,16.4) |  |
| Treatment | I | 5.6(4.1,7.1) | 0.305 | 14.9(7.5,22.3) | 0.489 |
|  | I+T | 5.6(5.0,6.1) |  | 15.0(12.9,17.1) |  |
|  | T | 6.2(5.5,6.0) |  | 14.0(8.6,19.5) |  |
| Therapies for patients with baseline LSMM | | | | | |
| LSMM-baseline | | 5.5(4.6,6.3) | 0.417 | 13.6(11.7,15.6) | 0.059 |
| I | | 3.7(2.1,5.3) | 0.524 | 9.8(5.0,14.6) | 0.594 |
| I+T | | 5.4(4.3,6.5) |  | 13.8(11.2,16.4) |  |
| T | | 6.2(4.6,7.7) |  | 12.6(2.4,22.8) |  |
| LSMM in I group | | 3.7(2.1,5.3) | 0.340 | 9.8(5.0,14.6) | 0.060 |
| LSMM in I+T group | | 5.4(5.0,6.1) | 0.365 | 13.8(11.2,16.4) | 0.272 |
| LSMM in T group | | 6.2(5.5,6.9) | 0.824 | 12.6(2.4,22.8) | 0.424 |
| Patients with LSMM after 3 months of different therapies. | | | | | |
| LSMM-3m | | 5.0(4.3,5.8) | 0.152 | 12.4(9.6,15.2) | 0.057 |
| I | | 4.6(2.8,6.3) | 0.477 | 14.0 | 0.391 |
| I+T | | 5.0(4.0,6.0) |  | 12.4(9.3,15.4) |  |
| T | | 6.0(4.8,7.3) |  | 7.3(4.1,15.2) |  |
| LSMM in I group | | 4.6(2.8,6.3) | 0.058 | 14.0 | 0.744 |
| LSMM in I+T group | | 5.0(4.0,6.1) | 0.371 | 12.4(9.3,15.4) | 0.085 |
| LSMM in T group | | 6.0(5.5,6.9) | 0.659 | 7.3(4.1,15.2) | 0.406 |
| Reduced ∆SMI with different therapies. | | | | | |
| Reduced ∆SMI | | 5.6(5.1,6.0) | 0.070 | 12.4(9.9,14.8) | 0.014 |
| I | | 4.8(3.1,6.5) | 0.785 | - | 0.132 |
| I+T | | 5.6(5.0,6.1) |  | 11.7(9.3,14.2) |  |
| T | | 6.1(5.1,6.0) |  | 12.6(6.0,19.2) |  |
| reduced ∆SMI in I group | | 4.8(3.1,6.5) | 0.835 | - | 0.298 |
| reduced ∆SMI in I+T group | | 5.6(5.0,6.1) | 0.186 | 11.7(9.3,14.2) | 0.001 |
| reduced ∆SMI in T group | | 6.1(5.7,6.4) | 0.172 | 12.6(6.0,19.2) | 0.732 |
| Progressing LSMM with different therapies. | | | | | |
| progressing LSMM | | 4.8(3.6,6.0) | 0.016 | 9.5(7.8,11.2) | <0.001 |
| I | | 4.6(1.7,7.4) | 0.661 | 6.8(4.2,9.5) | 0.643 |
| I+T | | 4.0(2.6,5.3) |  | 8.9(7.4,10.5) |  |
| T | | 6.0(4.7,7.3) |  | 12.6(6.6,25.4) |  |
| progressing LSMM in I group | | 4.6(1.7,7.4) | 0.344 | 6.8(4.2,9.5) | 0.268 |
| progressing LSMM in I+T group | | 4.0(2.7,5.3) | 0.039 | 8.9(7.4,10.5) | <0.001 |
| progressing LSMM in T group | | 6.0(4.7,7.3) | 0.338 | 12.6(6.6,25.4) | 0.981 |

Abbreviations:PFS (Progression-Free Survival); OS (Overall Survival); CI (Confidence Interval); TACE (Transarterial Chemoembolization); PVTT (Portal Vein Tumor Thrombosis); AFP (Alpha-Fetoprotein); BMI (Body Mass Index); ECOG-PS (Eastern Cooperative Oncology Group Performance Status); BCLC (Barcelona Clinic Liver Cancer); I (Immunotherapy); I+T (Targeted Therapy Combined with Immunotherapy); T (Targeted Therapy); LSMM (Low Skeletal Muscle Mass); ∆SMI (Change in Skeletal Muscle Index).

1. **Supplementary Table 7. Multifactorial COX regression of PFS and OS factors in Child-Pugh A patients.**

| Variables | OS | |  | PFS | |
| --- | --- | --- | --- | --- | --- |
|  | HR (95%CI) | P |  | HR (95%CI) | P |
| Age≥60 | / | / |  | 1.272(1.027,1.577) | 0.028 |
| Metastasis | / | / |  | 1.214(0.983,1.499) | 0.071 |
| PVTT | 1.426(1.086,1.874) | 0.011 |  | / | / |
| Reduced SMI | 1.142(0.849,1.536) | 0.382 |  | / | / |
| Progressing LSMM | 1.581(1.167,2.140) | 0.003 |  | 1.361(1.066,1.738) | 0.013 |

Abbreviations:COX (Cox Proportional Hazards Model); PFS (Progression-Free Survival); OS (Overall Survival); HR (Hazard Ratio); CI (Confidence Interval); P (P-value); SMI(Skeletal Muscle Index);LSMM (Low Skeletal Muscle Mass); PVTT (Portal Vein Tumor Thrombosis).

## Supplementary Figures


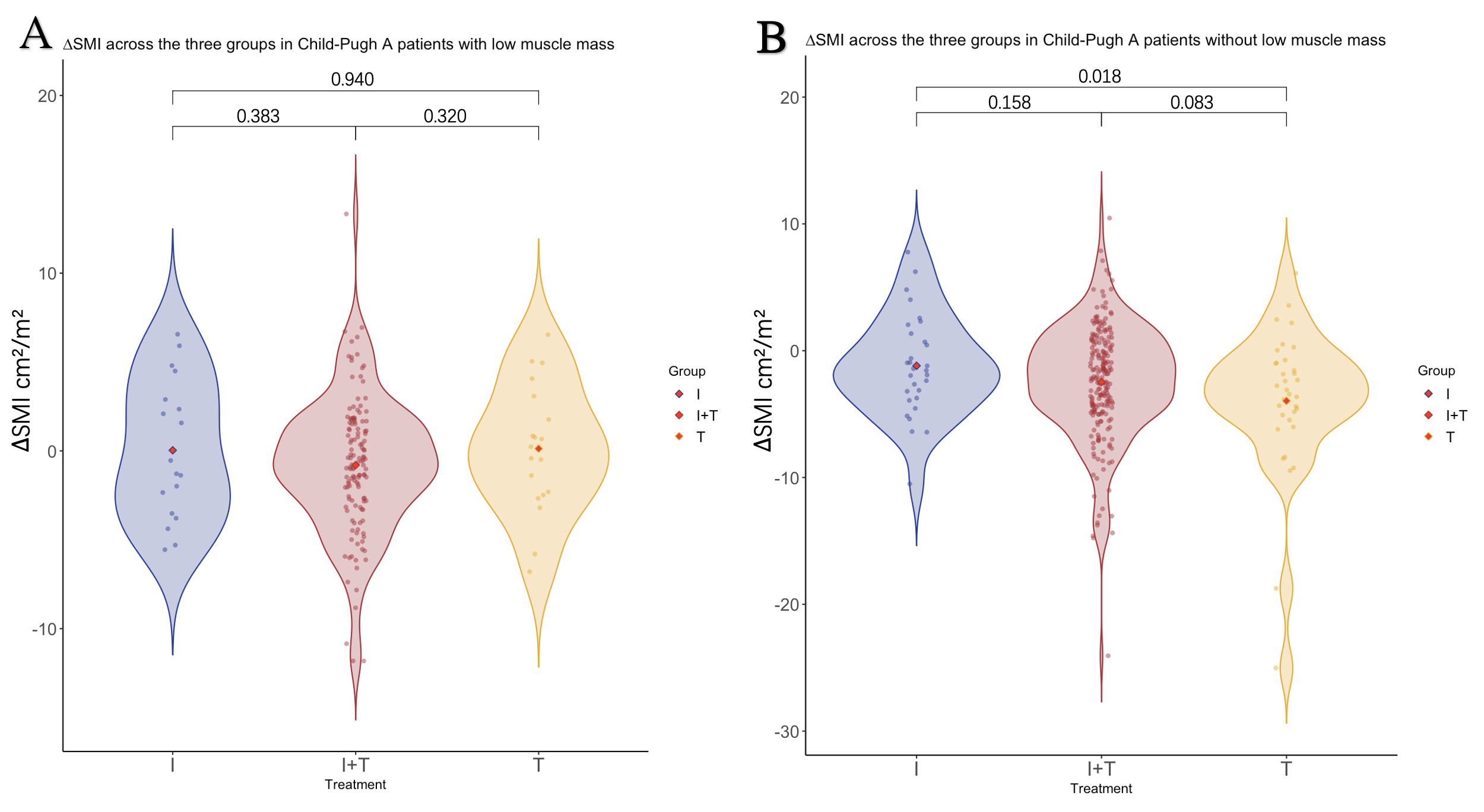


**Supplementary Figure 1.** **Differences in ∆SMI changes after 3 months of treatment across three therapeutic regimens in Child-Pugh A subgroup patients: (A) LSMM patients, (B) NLSMM patients. LSMM: Low skeletal muscle mass; NLSMM: Non-low skeletal muscle mass.**
